# Can Cold Atmospheric Plasma Be Used for Infection Control in Burns? A Preclinical Evaluation

**DOI:** 10.3390/biomedicines11051239

**Published:** 2023-04-22

**Authors:** Mahsa Bagheri, Maria von Kohout, Andreas Zoric, Paul C. Fuchs, Jennifer L. Schiefer, Christian Opländer

**Affiliations:** 1 Plastic Surgery, Hand Surgery, Burn Center, Cologne-Merheim Hospital, Witten/Herdecke University, Ostmerheimer Str. 200, 51109 Cologne, Germany; mahsa.bagheri@posteo.de (M.B.); maria.vonkohout@uni-wh.de (M.v.K.); fuchsp@kliniken-koeln.de (P.C.F.); schieferj@kliniken-koeln.de (J.L.S.); 2 Institute for Research in Operative Medicine (IFOM), Cologne-Merheim Hospital, Witten/Herdecke University, Ostmerheimer Str. 200, 51109 Cologne, Germany; andreas.zoric@googlemail.com

**Keywords:** burns, wound infection, cold atmospheric plasma hydrogen peroxide, Pseudomonas aeruginosa, biofilm, nitric oxide

## Abstract

Wound infection with Pseudomonas aeruginosa (PA) is a serious complication and is responsible for higher rates of mortality in burn patients. Because of the resistance of PA to many antibiotics and antiseptics, an effective treatment is difficult. As a possible alternative, cold atmospheric plasma (CAP) can be considered for treatment, as antibacterial effects are known from some types of CAP. Hence, we preclinically tested the CAP device PlasmaOne and found that CAP was effective against PA in various test systems. CAP induced an accumulation of nitrite, nitrate, and hydrogen peroxide, combined with a decrease in pH in agar and solutions, which could be responsible for the antibacterial effects. In an ex vivo contamination wound model using human skin, a reduction in microbial load of about 1 log_10_ level was observed after 5 min of CAP treatment as well as an inhibition of biofilm formation. However, the efficacy of CAP was significantly lower when compared with commonly used antibacterial wound irrigation solutions. Nevertheless, a clinical use of CAP in the treatment of burn wounds is conceivable on account of the potential resistance of PA to common wound irrigation solutions and the possible wound healing-promoting effects of CAP.

## 1. Introduction

The skin is the largest organ of the human body and serves as protection from the environment. It is not only a physical barrier against pathogens and mechanical injuries, but also serves to protect against unregulated loss of water and solutes. Furthermore, the skin provides a chemical/biochemical barrier with antimicrobial activity [1]. The skin of the human body is colonized by a diverse milieu of microorganisms, most of which are harmless, or even beneficial to the host. Colonization is determined by the nature and characteristics of the skin surface, which can vary widely, depending on topographic location, endogenous host factors, and exogenous environmental factors [2].

A burn occurs when the skin is exposed to a heat source. Here, the longer the exposure to heat and the higher the temperature, the greater and deeper the tissue damage. In addition, burn trauma can also result from freezing, electricity, chemicals, radiation, or friction [3]. After a burn, the skin and its barrier function are damaged or destroyed, sometimes over a large area. Deep second- or third-degree skin burns, if spontaneous healing occurs, heal slowly. The regeneration is dependent on the migration of keratinocytes from the surrounding uninjured skin toward the wound surface [3]. This process is also the basis for standard plastic surgery therapy for the rapid, permanent closure of burn wounds, in which donor split-thickness skin grafts are used to fill the wound [4].

Bacterial infections of burn wounds are among the most important and potentially serious complications that can occur in the acute period following injury [5,6]. Thermal destruction of the skin barrier and the reduced local and systemic immune responses of the body are critical factors that contribute to infectious complications in patients with severe burns [7,8,9]. Although the burned wound surface is primarily sterile, immediately after thermal injury, colonization often occurs within a few days [10,11]. Here, the second- or third-degree burn wound is a protein-rich environment consisting of non-vascularized necrotic tissue that provides a favorable niche for microorganism colonization and proliferation [12]. Most nosocomial infections of burn wounds result from multidrug-resistant Gram-negative bacteria [13]; *Pseudomonas* spp., *Staphylococcus aureus*, *Klebsiella* spp., *Proteus* spp., *Enterococcus* spp., and *Escherichia coli* are the pathogens that can be isolated from infected burn wounds [14,15]. In particular, *Pseudomonas* spp. was detected here most frequently. Sepsis induced by wound infection is associated with high mortality and poses a threat to patient survival [13].

In particular, infections with *Pseudomonas aeruginosa* (PA) significantly increase mortality in burn patients, especially when acquired nosocomially. Here, MDR (multi-drug resistant) PA has increasing importance as a main cause of death in burn patients, due to the high occurrence of MDR bacteria in burn centers [16,17]. In addition, burn wounds trigger PA to produce pathogenic factors and biofilms, which, in turn, can cause delays in healing [18]. Hence, wound infections with PA significantly increase the length of hospital stays, the number of days on mechanical ventilation, the number of surgical procedures, and the amounts of blood products used [19].

PA is able to survive in almost any environment on account of its low nutrient requirements. It is named for its characteristic green coloration in purulent wound infections, which is caused by the blue-green pigment pyocyanin and the dye fluorescein. Infections can be easily identified by the characteristic, penetrating sweet aromatic odor, because of the formation of 2-aminoacetophenone. In addition, for the identification of PA and differentiation from other pathogens, its fluorescent property can be used [20,21,22]. PA, like all Gram-negative bacteria, has an outer membrane that is a diffusion barrier for most antibiotics. Antibiotics can only enter the bacterium through narrow and impermeable porins, which is why most antibiotics cannot penetrate the bacterium. Hence, PA is naturally resistant to many antibiotics [20]. In addition, PA possesses other intrinsic resistance mechanisms to antibiotics, such as the expression of efflux pumps that expel antibiotics from the cell and the production of antibiotic-inactivating enzymes like β-lactamases [23,24,25].

Infections of burn wounds caused by *P. aeruginosa* are treated systemically with antibiotics and locally with the antiseptics mafenide acetate and citric acid [26,27,28]. However, mafenide acetate has—apart from an antimicrobial effect—cytotoxic properties, and can cause metabolic acidosis and delayed wound healing [29,30,31]. As an addition to and/or alternative for topical treatment, 3% citric acid is used to treat burn wounds infected with PA [32]. Very often, conservative therapy attempts are unsuccessful, so that a distressing and aggressive surgical debridement is necessary as a last therapy option [33,34].

Cold atmospheric plasma (CAP) is a physical low-temperature plasma generated at atmospheric pressure, containing a highly reactive mixture of UV radiation, reactive oxygen, and reactive nitrogen species (ROS/RNS), such as O_3_, O_2_^−^, OH^−^, NO_2_, and NO, and shows, in general, a broad-spectrum antimicrobial efficacy [35]. There are many devices that produce different types of CAP. A distinction is made between direct, indirect, and hybrid CAPs. Direct CAP is generated between two electrodes by energizing the surrounding air, where the body or tissue can act as a counter-electrode. The CAP is, therefore, created between the electrode and skin/wound and no carrier gas is required. An example of a direct CAP source is dielectric barrier discharge (DBD). Here, the electrodes are enclosed by a non-conductive layer, a dielectric, and the discharges take the form of many small micro-discharges. Indirect CAPs are generated by plasma jets, which form the plasma between two electrodes within the device and are transported to the target via a carrier gas, normally with the noble gases argon or helium. Hybrid CAPs represent a combination of direct and indirect plasma. These are generated in the same way as direct plasma, but have current-free properties on account of a grounded grid electrode. An example of a hybrid plasma source is the corona discharge. Unlike DBD, there is no air space between the barrier and the counter electrode [35,36,37,38].

As a kind of direct plasma source, the PlasmaOne cold plasma device does not require any gas supply with noble gases to generate therapeutically effective CAPs, only the ambient air. The control center supplies the converter with a direct current, which is converted into high frequencies. Inside the treatment probe made of glass—which is filled with helium—these high frequencies are conducted via the ionized helium molecules to the tip of the probe and an electric field is now formed between the patient’s skin and the treatment probe, leading to the ionization of the atoms and molecules of the ambient air. This generates the therapeutically effective CAP between the skin/wound and the probe surface. The predominant reactive species found in the generated CAP are NO, NO_2_, and O_3_, according to information provided by the company [39].

Normally, CAPs do not cause thermal damage to the tissue, which significantly expands the possible applications of the plasma. In medical applications, CAP therapies are sometimes used in dermatology and dentistry, with distinct CAP devices for treating acute and chronic wounds [4]. However, the evidence base on the efficacy of cold plasma is still narrow and difficult to assess. The individual studies with different generation methods and CAP devices are difficult to compare. In addition, the effectiveness of the therapy also depends on the dose and duration of application [40]. Furthermore, although CAP shows good antibacterial efficacy and promising effects on biofilms on more or less dry biomaterials in vitro, it seems that the antibacterial effects in vivo or under wet conditions are much less pronounced and possibly not clinically relevant [41,42].

Therefore, in this present study, we have examined the antimicrobial efficacy against PA of a direct CAP, generated by the PlasmaOne^®^ device, as an alternative option for the treatment of (burn) wound infections, using a quantifiable human skin wound contamination model, which is closer to the clinical reality than standard microbiology assays [43,44].

## 2. Materials and Methods

### 2.1. Cold Atmospheric Plasma (CAP) Source and CAP Treatment

For CAP treatments, the medically accredited device Plasma One (MEDICAL SYSTEMS GmbH, Nassau, Germany) was used. This device is battery-powered; CAP is generated by a floating electrode dielectric barrier discharge (FE-DBD). To generate CAP, the device does not require any admixture of noble gases like argon or neon, only the ambient air. The energy control center supplies the converter with a direct current and this is converted into high frequencies (Hƒ), which are conducted to the tip of the treatment probe via the noble gases within it. Through an electrode, the patient/sample has contact with the energy control during treatment and an electric field is formed between the patient’s skin and the treatment glass probe, which leads to the ionization of the atoms and molecules of the ambient air, generating a direct CAP in this space between probe and tissue/sample.

For our experiments, the device was conducted in Mode 5, which is the maximal power level, and the treatment glass probe PS30 with an outer tip diameter of 34 mm (power: 5 W, repetition frequency: 1220 Hz, converter pulse width: 10 μs). The CAP was applied at a distance of 1–2 mm between the tip of the treatment glass probe and the sample (agar plate, skin wound). At this distance, the activity monitor of the device is activated and the CAP generation is continued [45].

### 2.2. CAP-Induced Accumulation of Nitrite and Nitrate

On casein/soy peptone agar plates (diameter 10 cm), 100 µL TSB was spread and incubated for 20 min at 37 °C prior to CAP treatment. The CAP treatment glass probe was mounted on a stand and an agar plate was placed centrally on the laboratory lifting platform, which was used to slowly lift the agar plate to the probe tip until stable ignition and CAP generation were formed. This was normally the case with a distance of 1–2 mm between the probe tip and the agar surface. This approach avoided contact of the probe with the agar surface. The agar was grounded by a wire and treated with CAP for 0, 30, 60, 150, 300, or 600 s.

**Figure 1:**
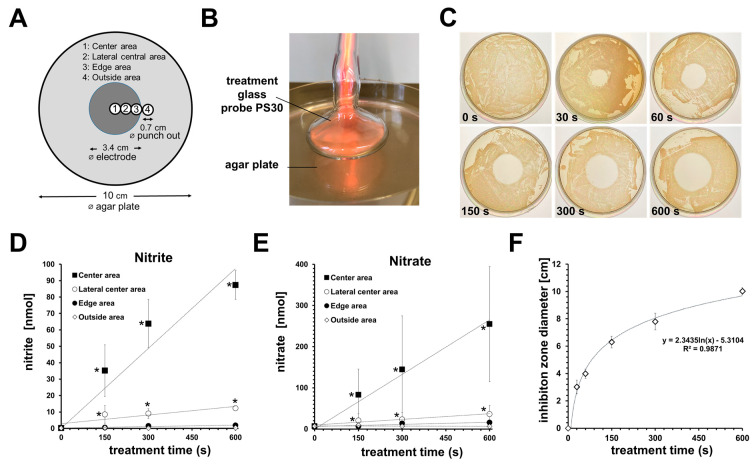
CAP treatment showed antibacterial efficacy against *Pseudomonas aeruginosa* and induced nitrite/nitrate accumulation. (**A**) Overview of the CAP treated area and sample locations within the agar plate. (**B**) Shown here is a representative photograph of a CAP treatment of an agar plate. (**C**) Exemplary photographs of agar plates cultivated with *Pseudomonas aeruginosa*, with inhibition zones induced by different CAP treatments (as indicated). (**D**) shows the amount of nitrite and (**E**) nitrate found in the punch biopsy samples taken from the different areas of the agar plates after CAP treatment as indicated (n = 4; * *p* < 0.05 compared to untreated controls). (**F**) Given are the mean values of the measured diameter of the CAP-induced inhibition zones (n = 3; * *p* < 0.05 compared to untreated controls).

After treatment, certain areas of the agar plates were punched out (diameter 4 mm), as seen in Figure 1A, dissolved in 950 µL phosphate-buffered saline (PBS, pH 7.4), homogenized for 1 s by an Ultra turrex T8 disperser (IKA, Staufen, Germany), and centrifuged at 1000× *g* for 1 min. The supernatants obtained were used for nitrite/nitrate measurements by an iodine/iodide-based and vanadium (III) chloride/hydrochloric acid-based assay using a NO analyzer (CLD 88, Ecophysics, Munich, Germany), as described elsewhere [46]. These experiments were performed four times independently.

In another experimental setup, 120 µL TSB containing PA was distributed in a circle (diameter: ~2 cm) on a microscope slide (Menzel-Gläser, Thermo Scientific, Waltham, MA, USA). The slide was mounted on a metal laboratory jack and the TSB drop that was spread was connected with the electrode of the CAP device with a thin wire, which was immersed in the liquid at the edge. Using the lifting platform of the laboratory jack, the TSB drop was placed centrally under the tip of the treatment glass probe at a distance of 1–2 mm and CAP was applied for 0, 60, 150, 300, or 600 s. The TSB was aspired and transferred with a pipette into a cooled 1.5 mL centrifugation tube (Eppendorf, Hamburg, Germany), and the amounts of nitrite and nitrated were analyzed.

### 2.3. Measurement of CAP-Induced Hydrogen Peroxide

The concentration of hydrogen peroxide (H_2_O_2_) in CAP-treated TSB was determined using the titanium oxide oxalate method, as described elsewhere [46]. In brief, a stock solution of potassium titanium oxide dihydrate (0.1 M) with 2 M sulfuric acid was freshly prepared. The CAP treatment was conducted analogously to the samples for the nitrite/nitrate measurements with 120 µL TSB spread on a microscope slide (see above).

Directly after CAP treatment, 100 µL of the TSB was mixed with 100 µL of the titanium oxide stock solution and transferred to a 96-well plate. The absorbance at 400 nm was measured using a photometer (Epoch II, BioTek, Winooski, VT, USA). The concentrations of H_2_O_2_ were calculated using calibration curves with known H_2_O_2_ concentrations (0–500 μM in TSB). In preliminary experiments, the presence of high nitrite/nitrate concentrations (both 2 mM) and low pH values (5.5 and 6.4) had no significant effects on calibration curves.

### 2.4. Determination of CAP-Induced Changes of pH Value

The pH values of the TSB and skin surface w/o CAP treatment were measured using a pH meter equipped with a flat pH electrode (PH CHECK F, Th. Geyer, Lohmar, Germany).

### 2.5. Determination of CAP-Induced Antibacterial Effects on P. aeruginosa

#### 2.5.1. Pseudomonas Strain and Culture Conditions

The bacterial culture of *Pseudomonas aeruginosa* (PA) used was provided by the Leibniz Institute DSMZ—German Collection of Microorganisms and Cell Culture (batch No.: 0411). For the experiments, the subculture II of PA was used. Prior to the experiments, a master plate was prepared using the cryopreserved PA sample and cultivated on tryptone soya agar (TSA) plates (Sigma-Aldrich, Munich, Germany) for 24 h and was subsequently kept at 7 °C for a maximum of 2 weeks. A bacterial strain was prepared by picking a colony from the master plate 24 h before CAP treatment and incubated in 25 mL tryptone soya broth (TSB) culture medium at 37 °C. Using a photometer to measure the absorbance at 600 nm (Epoch II, BioTek, Winooski, VT, USA), the PA solution was diluted to a 0.5 McFarland standard, which corresponds to approximately 1.5 × 10^8^ CFU/mL, and then further diluted in TSB to the specific concentration required for the experiments.

#### 2.5.2. Determination of CAP-Induced Inhibitory and Bactericidal Effects on *P. aeruginosa*

**Figure 2:**
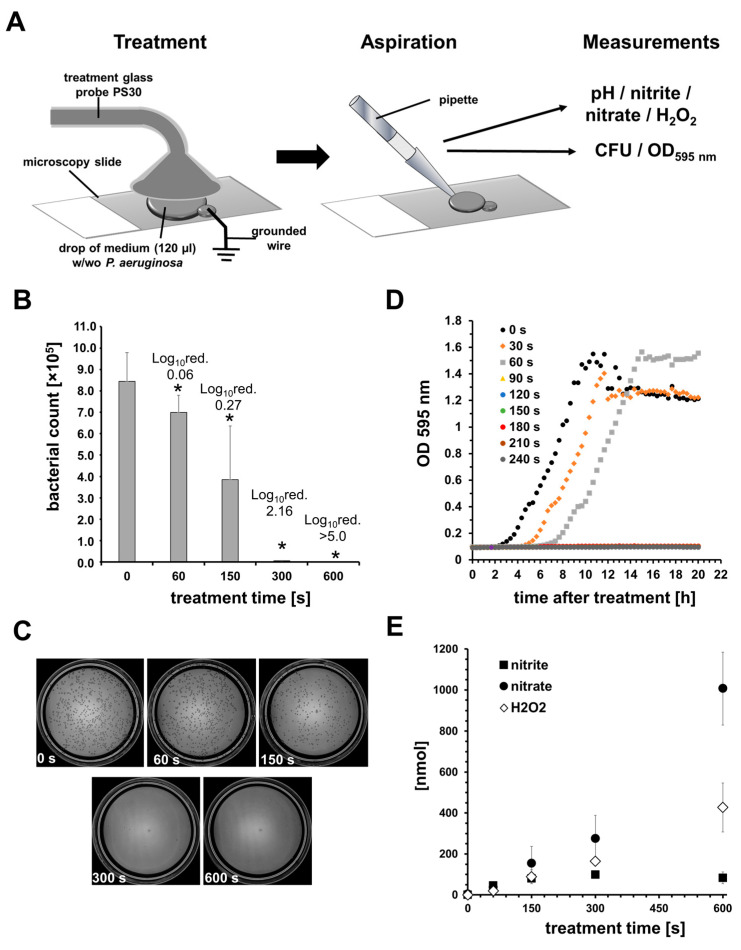
CAP treatment can reduce bacterial load in small volumes of medium. (**A**) Overview of the experimental procedure. (**B**) Given are the mean ± SD values of the number of *Pseudomanas aeruginosa* (PA) found after CAP treatments of small volumes of PA-containing medium (1 × 10^6^/120 µL). n = 3; * *p* < 0.05 compared to untreated controls. (**C**) Shown are representative photographs of the respective agar plates after CFU assays (10^4^ dilution). (**D**) Exemplary measurements of the optical density at 595 nm of PA-containing media further cultivated after different CAP treatments (as indicated). (**E**) Amounts of nitrite, nitrate, and hydrogen peroxide (H_2_O_2_) found in small volumes of medium after CAP treatment (n = 3).

Analogous to the determination of nitrite/nitrate, 120 µL TSB containing PA (1.8 × 10^6^ CFU) was distributed in a circle (diameter ~2 cm) on a microscope slide (Menzel-Gläser, Thermo Scientific, Waltham, MA, USA) mounted on a metal laboratory lifting platform, and connected with the electrode of the PlasmaOne CAP device with a thin wire (see Figure 2A). The microscope slide was lifted slowly up to the treatment probe until a distance of 1–2 mm from the probe and CAP was applied for 0, 30, 60, 90, 120, 150, 180, 210, or 240 s. Any contact of the probe with the liquid was avoided. Adapted from common assays to determine minimum inhibitory concentrations, directly after treatment, 100 µL of the CAP-treated TSB drop was transferred into a 96-well plate with a flat bottom and transparent lid (TC-Plate, Sarstedt, Nürmbrecht, Germany). The 96-well microplate was subjected to absorbance readings using a microplate spectrophotometer (EPOCH II, Biotek, Winooski, VT, USA) and read at a wavelength of 595 nm for 20 h, with 20 min intervals and agitation of 10 s. The growth curves obtained from the OD measurements were evaluated to determine the minimum inhibition CAP treatment time. Analogously, 120 µL TSB containing PA (1.8 × 10^6^ CFU) was treated with CAP (0, 60, 150, 300, 600 s). Here, 100 µL of the CAP-treated TSB was serial diluted for the CFU assay; thus, 100 µL of the dilution was plated on TSA plates and incubated for 24 h at 37 °C to determine the bacterial survival rate by counting colonies. Each single experiment was performed independently three times in duplicate.

#### 2.5.3. Agar Inhibition Zone Assays

On TSA plates (diameter: 10 cm), 100 µL TSB containing 1.5 × 10^7^ CFU was spread and incubated for 20 min at 37 °C prior to CAP treatment. The CAP treatment glass probe was mounted on a stand and the agar plate was placed centrally on a laboratory lifting platform and lifted slowly up to probe tip until a distance of 1–2 mm between probe tip and agar surface. The agar was grounded by a wire and treated with CAP for 0, 30, 60, 150, 300, or 600 s. Thereafter, agar plates were incubated overnight at 37 °C before photos were taken. The diameter of the inhibition zones was determined with the help of the ImageJ^®^ software (v. 1.53k) [47]. These experiments were performed independently three times.

#### 2.5.4. Skin Wound Contamination Model

Human skin specimens from four patients aged between 27 and 49 (mean age 42) were obtained from abdominoplastic surgeries provided by the Clinic for Plastic Surgery, Hand Surgery, and Burn Center at the Cologne-Merheim Hospital. The use of human donor skin was approved by the ethics committee of the University of Witten/Ethics Herdecke’s Committee (Votum No. 15/2018), and all experiments were conducted in compliance with the Declaration of Helsinki Principles. Patient consent was obtained for research purposes. The donor skin specimens were transported postoperatively in a sterile container on ice to the laboratory. Here, the skin was washed with sterile NaCl and incubated for one minute with 70% ethanol (Carl Roth, Karlsruhe, Germany) before further processing. In a previous study, we established a wound skin model [44], which was modified for this study.

**Figure 3:**
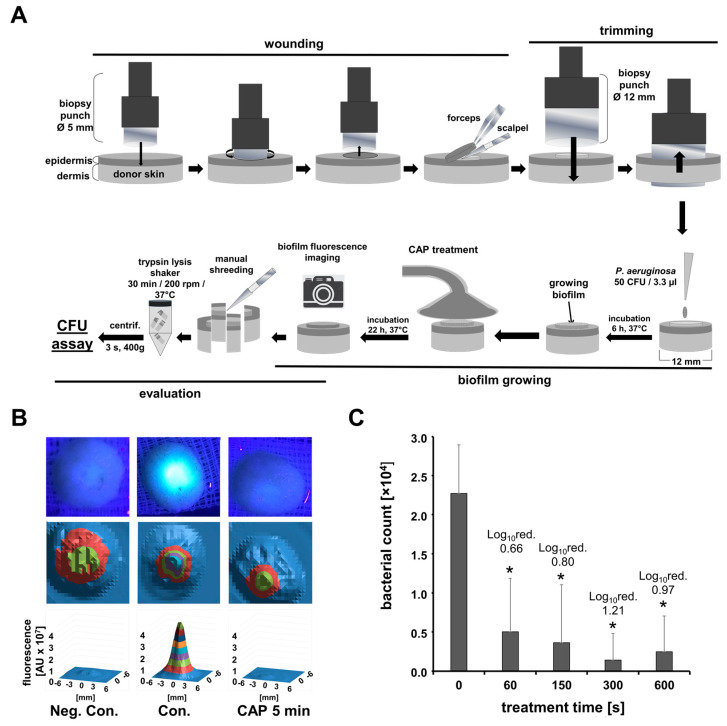
CAP treatment reduced bacterial burden in a skin/wound model. (**A**) The experimental procedure of the skin/wound model, CAP treatment, and evaluation of the bacterial burden is illustrated. (**B**) Exemplary photos of black light-induced fluorescence of contaminated (Neg. Con = uncontaminated) wounds in the skin/wound model after different treatments, as indicated. The fluorescence signal of the bacterial biofilm was evaluated further using a multiplate photometer. (**C**) Given are the means of the relative bacterial counts (to the respective untreated control) and the calculated Log_10_ reduction after different CAP treatments (0–10 min), assessed by CFU assay after trypsin lysis of the skin samples (n = 4; * *p* < 0.05).

Standardized skin samples were prepared, as shown in Figure 3A. First, wound areas 5 mm and 1 mm in depth were prepared using a biopsy punch (5 mm, Acuderm Inc., Fort Lauderdale, FL, USA) for a superficial cut of the epidermis, which was then carefully removed with a scalpel and forceps. Second, using a larger biopsy punch (12 mm; Acuderm Inc., Fort Lauderdale, FL, USA), round skin samples were punched around the 5 mm wounds. These round skin samples were placed onto sterile gauze pads (1.5 × 1.5 cm) in single culture plates (35 mm, Greiner, Frickenhausen, Germany) and 5.5 mL of cell culture medium (DMEM w/o phenol red; 1.0 g/L glucose, with 10% fetal calf serum, PAN Biotech, Aidenbach, Germany) was added to each plate. Finally, defined volumes of 3.3 μL TSB containing PA (50 CFU) were applied onto each wound area and the skin/wound samples were incubated for 6 h (37 °C, 5% CO_2_) prior to CAP treatment, which were performed in duplicates. The CAP treatment glass probe was mounted on a stand and the culture plate with the skin sample was placed on a laboratory lifting platform with a distance of 1–2 mm between probe tip and skin surface, whereas the wound was in the central area of the probe tip. The skin was grounded by a wire, which was immersed in the medium, and treated with CAP for 0, 30, 60, 150, 300, or 600 s.

Subsequently, the skin samples were incubated for 16 h at 37 °C. After incubation, the duplicates were pooled and cut into small pieces, transferred into centrifugation tubes (50 mL, Greiner, Frickenhausen, Germany), and enzymatically digested in 5 mL of 0.25% trypsin/HBSS solution (PAN Biotech, Aidenbach, Germany) in an incubator on a shaker (CO_2_-resistant, 3 mm Orbit, Thermo Fisher Scientific, Waltham, MA, USA) for 30 min at 150 rpm. After a centrifugation of 3 s, 100 μL of the solution was plated on agar plates to determine the bacterial survival rate (CFU/mL). These experiments were performed four times independently with different skin specimens. Before cutting and enzymatic digestion, we used a commercially available LED black light torch (395 nm, Bestsun, Jiaxing, Zhejiang, China) to capture exemplary photos of the bacterial biofilm fluorescence on the skin/wound samples under black light illumination in a dark room. Furthermore, we measured the biofilm fluorescence using a multiplate reader (VictorNivo, PerkinElmer, Waltham, MA, USA). Thereafter, the skin/wound samples with or without PA contamination were transferred to a 12-well cell culture plate (Greiner, Frickenhausen, Germany) without medium for fluorescence intensity scans by top measurement (ext. 405 nm; em. 530 nm; rows and columns 24 × 24 bi-directional; row/column range 12 mm; measurement time 100 ms; flash energy 10 mJ, PMT HV 500 V; excitation spot size 0.5 mm; emission spot size 1.0 mm).

### 2.6. Statistic

The software GraphPad Prism Version 8.4.3 (San Diego, CA, USA) was used for statistical analyses; significant differences with a *p*-value of <0.05 were evaluated using one-way ANOVA. Each experiment was independently performed 3–4 times.

## 3. Results

### 3.1. CAP Treatment Induced Physical/Chemical Changes

#### 3.1.1. CAP Treatment Locally Increased the Nitrite and Nitrate Content of Agar

Our experiments showed that plasma treatment led to a local accumulation of nitrite and nitrate in the agar. The degree of enrichment depended on the localization under the treatment probe during CAP treatment. The accumulation in the area below the center of the probe was significantly higher (~10×) than in the lateral areas (see Figure 1). In the center, ~250 nmol of nitrate and ~85 nmol of nitrite were found in an area of 0.385 cm^2^ after a 10 min CAP treatment. With respect to the volume of the agar, we estimate that the concentrations of nitrite obtained in this spot were around 1.75 mM, and of nitrate, around 5 mM.

#### 3.1.2. CAP Treatment Increased the Nitrite, Nitrate, and H_2_O_2_ Contents in Medium

The exposure of liquid—in our case, TSB—to CAP led a linear increase in nitrate and H_2_O_2_ concentrations, as shown in Figure 2E. In total, around 1000 nmol of nitrate was found in 120 µL of TSB after a 10 min CAP treatment, which equals a concentration of ~8.3 mM. The amount of accumulated H_2_O_2_ was around 450 nmol, which is equal to a concentration of ~3.5 mM. The amount of nitrite did not increase linearly. Here, after CAP treatment for 10 min, the nitrite amount/concentration of ~83 nmol/690 µM was lower than after a 5 min CAP treatment, which achieved ~98 nmol/816 µM.

#### 3.1.3. CAP Treatment Induced Acidification

The surface pH value 7.0 in the middle of a TSA plate was decreased by a 5 min CAP treatment down to 4.9 and after 10 min to 4.4. When dealing with small volumes of TSB (120 µL) spread on a microscopy slide, a pH value of 5.6 and 4.7 could be found after CAP treatment of 5 min and 10 min, respectively. The surface pH value of intact human skin was, in our experiments, around 5. After a single CAP treatment longer than 150 s, the skin surface was significantly acidified, achieving pH values between 1 and 2 (Figure S1).

### 3.2. CAP Induced Antibacterial Effects

With regard to a possible antibacterial effect of the CAP treatment, our experiments showed that this depended on the treatment time and on the type of test system. When a PA-contaminated agar plate was CAP-treated, a clear inhibition zone could already be seen after 30 s of CAP exposure, which became larger with increasing treatment time. After 10 min of CAP treatment, the inhibition zone extended across the entire area covered by the treatment probe (Figure 1C,F).

When treating small volumes in a flat volume of bacterial suspension with CAP, a relevant bactericidal effect of CAP was only detected after 300 s of treatment time. An almost complete elimination of the bacterial load in a small and flat volume of liquid could be observed in our experiments after a 10 min CAP treatment (Figure 2B,C). In addition, an inhibition of bacterial growth was observed after a treatment time of 90–120 s, when PA had been further cultivated in the treatment medium after CAP treatment (Figure 2D). In preliminary tests with larger and higher volumes, we could not detect any significant antibacterial efficacy even after longer treatment times.

A reduction in the number of bacteria by the CAP treatment was observed in the more realistic skin wound contamination model as well. Here, a 1 min treatment resulted in about a 65%, and a 2.5 min treatment resulted in an 80% lower bacterial load compared to the untreated control. Only after 5 and 10 min of treatment, a reduction in the bacterial count of over 90% could be achieved. Here, the 10 min treatment was not more effective than the 5 min treatment. The reduction in the bacterial load was also associated with a lower bacteria/biofilm fluorescence signal in the wound area one day after CAP treatment.

## 4. Discussion

The Gram-negative bacterium *Pseudomonas aeruginosa* is one of the most commonly isolated organisms from infected burn wounds [14,15]. The ability of PA to maintain persistent infections through biofilm formation can result in significant delays in the healing of burn wounds. The main goal of treating infected wounds in burn patients is to reduce the bacterial load and prevent complications like sepsis and death and allow the skin to heal without further complications [16,18]. Some CAP systems showed antibacterial activity, albeit moderate, against a wide range of pathogens [48,49,50].

Therefore, in the present study, we investigated the antimicrobial efficacy of CAP generated by the PlasmaOne device, which showed promising antibacterial effects on *Staphylococcus aureus* and also PA in in vitro studies, and could be an alternative option for the treatment of (burn) wound infections caused by PA [51,52]. However, it was observed that PA was more resistant against CAP than *Staphylococcus aureus*, when contaminated solid surfaces (agar plates) were treated [52].

In various test systems, we were also able to demonstrate an antibacterial effect of plasma treatment with this CAP source. Depending on the test system, the antibacterial efficacy varied significantly. With a low volume of liquid and a flat surface, such as a thin film of liquid on an agar plate, even short treatment times were sufficient to achieve a bacteria-free zone. If the liquid volume was somewhat larger and higher, as in the suspension experiment with a spread droplet containing PA, longer treatment times were necessary to achieve a significant reduction in the bacterial load. With the larger liquid volumes (1 mL/6-well cell culture dish) used in preliminary experiments, we could not detect a significant reduction in the number of bacteria after CAP treatment. In the skin wound model, the liquid volume was low and flat, but the wound surface was uneven. In addition, there was biofilm formation, which can protect the bacteria against CAP. Additionally, under these conditions, longer CAP treatments up to 10 min achieved only a 90% reduction. In comparison, the 15 min application of antibacterial Polyhexanide-containing wound irrigation solutions (Pronotosan) achieved a bacterial reduction of over 98% in a similar assay (data not published).

Hence, the use of compresses fully saturated with antibacterial wound irrigation solutions appears to be a much easier, faster, and also more effective way for the prophylaxis and treatment of wound infection in everyday clinical practice than plasma treatment. In particular, for more extensive burn wounds, CAP treatment would be very time-consuming. Another shortcoming of plasma treatment is the lack of homogeneity of the plasma distribution over a wide area. Our experiments showed that on smooth surfaces, the bioactive plasma was more effective in the center under the treatment electrode, where it causes an accumulation of nitrite and nitrate and reduced bacterial growth after a short treatment, while under the margin area of the electrode a longer CAP treatment (5–10 min) was required, probably mediated indirectly by diffusion of CAP-treated medium. On the one hand, in the case of uneven surfaces—such as wound edges—it can be observed that direct plasma preferentially ignites at the higher points, thereby reaching the intact skin at the wound edge rather than the wound surface itself. On the other hand, treatment of the wound margin could ensure that skin bacteria do not migrate from the skin area into the wound. Here, the accumulation of nitrite and nitrate as well as a strong decrease in the skin pH value at the wound edge may lead to a chemical barrier for many pathogenic bacteria.

In addition to the antibacterial effects, other possible wound healing effects of plasma treatment should not be underestimated. Via acidification of the wound area and enrichment with nitrite/nitrate as described above, it is known that some types of CAPs produced by dermal barrier discharge (DBD) can induce a local increase in microcirculation [53,54].

Furthermore, it is conceivable that many radicals produced by CAP may positively influence the cell physiology of fibroblasts and keratinocytes at lower doses, as observed in some studies using other types of CAPs [46,55].

However, the dose makes the poison, and, in many studies, toxic effects on cells induced by CAP, particularly at higher doses, could be observed [42,56,57].

## 5. Conclusions

Our results indicate that CAP therapy alone, using the PlasmaOne device, is probably not sufficient for the treatment of PA-contaminated burn wounds. In comparison with antibacterial wound irrigation solutions, the antibacterial efficacy that was observed was significantly lower; hence, CAP treatment of larger PA-infected burns would be very time-consuming. Nevertheless, additional CAP therapy with PlasmaOne could potentially provide benefits. In particular, apart from the better antibacterial efficacy against other common wound pathogens compared to PA [52], CAP-induced effects, such as the observed acidification of the wound area, could have a positive impact on wound healing [58]. Furthermore, possible improvement of local microcirculation and stimulation of skin cells—as observed with other CAP devices [53,54,59]—may have beneficial effects on the healing process. However, further clinical and experimental studies are necessary to verify this assumption.

## Data Availability

The data that support the findings of this study are available from the corresponding author upon reasonable request.

## Supplementary Materials

The following supporting information can be downloaded at: https://www.mdpi.com/article/10.3390/biomedicines11051239/s1, Figure S1: CAP-induced acidification
