# Supplementary material for: Can Cold Atmospheric Plasma Be Used for Infection Control in Burns? A Preclinical Evaluation
Source: Biomedicines. 2023 Apr 22;11(5):1239. doi: 10.3390/biomedicines11051239 (PMC10215252; doi:10.3390/biomedicines11051239)
Supplement: Supplementary file 1 [file biomedicines-11-01239-s001.zip › biomedicines-2311222-supplementary.docx]

Supplementary Materials

Can Cold Atmospheric Plasma Used for Infection Control in Burns? A Preclinical Evaluation

Mahsa Bagheri, Maria von Kohout, Andreas Zoric, Paul C. Fuchs, Jennifer L. Schiefer and Christian Opländer


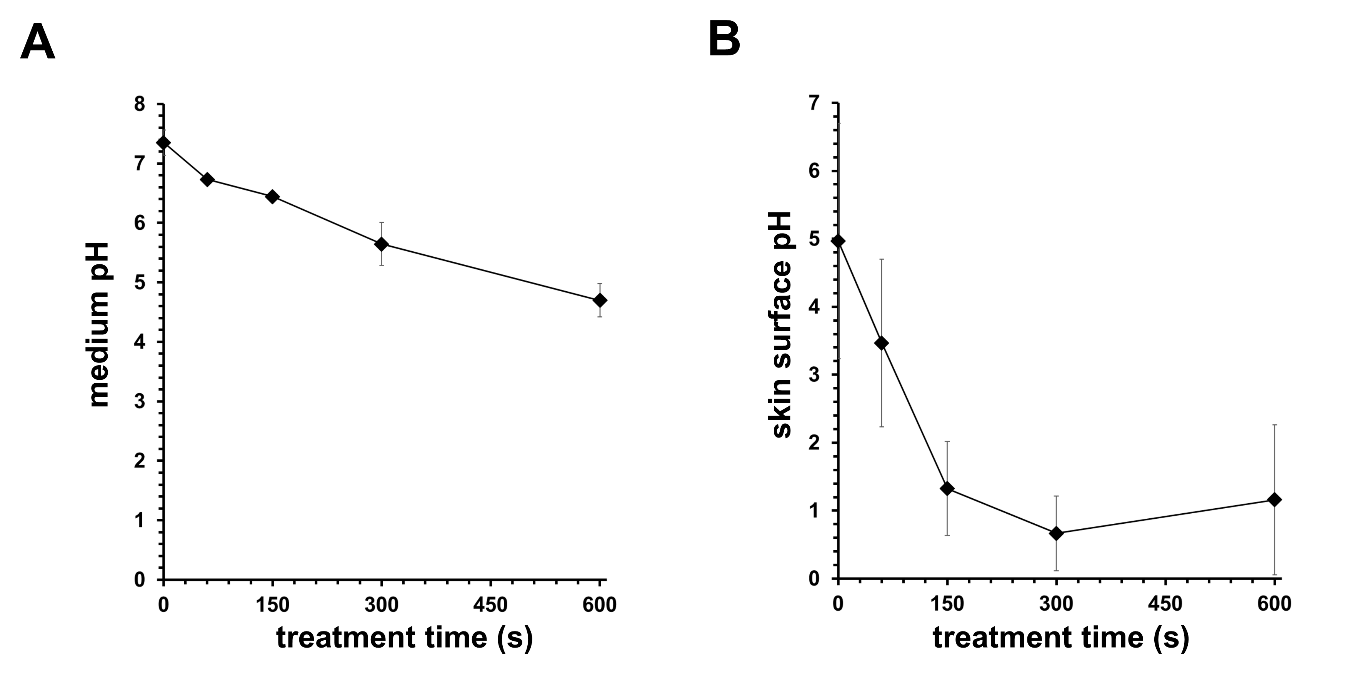


Figure S1: CAP-induced acidification. **A)** Culture medium (TSB; 120 µl) spread on a microscopy slide was treated by CAP (as indicated) and pH value was measured directly after treatment (n=3). **B)** Vitale human skin samples were treated with CAP (as indicated) and pH value of the skin surface was measured directly after treatment (n=4).
